# The Effects of Interventions on Health‐Related Quality of Life of People Living With Cardiovascular Disease: A Systematic Review

**DOI:** 10.1111/jocn.17770

**Published:** 2025-04-28

**Authors:** Amineh Rashidi, Lisa Whitehead, Lisa Newson, Helen Walthall, Clare Van Miert, Vivien Kemp, Ian Jones

**Affiliations:** ^1^ School of Allied Health (Nursing) The University of Western Australia Crawley Western Australia Australia; ^2^ School of Nursing and Midwifery Edith Cowan University Joondalup Campus Joondalup Australia; ^3^ School of Psychology Faculty of Health, Innovation, Technology and Science Liverpool John Moores University Liverpool UK; ^4^ Oxford University Hospitals, NHS Foundation Trust, Corporate Nursing Office, John Radcliffe Hospital Oxford UK; ^5^ School of Nursing and Advanced Practice Liverpool John Moores University Liverpool UK

**Keywords:** cardiovascular disease, cardiovascular rehabilitation, health‐related quality of life, intervention

## Abstract

**Aim:**

To synthesise the evidence from randomised controlled trials on the effectiveness of interventions to improve health‐related quality of life in people living with cardiovascular disease.

**Design:**

Systematic review and narrative synthesis.

**Data Sources:**

A systematic search of eight databases was conducted to identify relevant papers that were published in English and peer‐reviewed.

**Review Methods:**

The titles and abstracts of the articles were screened by two independent reviewers. The remaining articles underwent full text screening, followed by quality appraisal conducted by two independent reviewers.

**Results:**

This review included 13 studies. The intervention in all studies was cardiac rehabilitation. In spite of this, the studies used different measures of health‐related quality of life (HRQL) which prevented the conduct of a meta‐analysis. Four themes were identified in the reported findings.

**Conclusion:**

Understanding the specific aspects of cardiac rehabilitation that are related to the improvement of physical and mental HRQL of people living with cardiovascular disease requires further consideration and then incorporation into nursing plans and nursing interventions to enhance health outcomes.

## 1 Introduction

Cardiovascular diseases (CVD) include a range of conditions affecting the heart and blood vessels and include coronary heart disease, heart failure, stroke, and peripheral artery disease (AIHW 2022). The World Health Organisation (WHO) considers CVD the leading cause of death globally, with 75% of deaths occurring in low‐and middle‐income countries (World Health Organisation 2021). It is reported that CVD was the underlying cause of 25% of all Australian deaths in 2019 and accounted for almost 13% of the total disease burden in 2018, of which 76% resulted in the loss of life due to premature death, while 24% of the burden accounts for years of living with the disease (AIHW 2021). The World Heart Federation (2023) reports that deaths from CVD have increased by 60% globally over the last 30 years, with a jump globally from 12.1 million in 1990 to 20.5 million in 2021. The disease burden of CVD is often estimated using measures of health‐related quality of life (HRQL). HRQL refers to the subjective assessment of individuals' well‐being and ability to perform social roles and physical and psychological functions (Guyatt et al. 2007; Lin et al. 2013). Episodes of CVD have complex and impairing effects on HRQL among patients and are positively correlated with higher readmission and mortality rates (Conradie et al. 2022).

HRQL can be considered one of the most important indicators of treatment effectiveness; therefore, achieving an optimal HRQL is crucial to people living with CVD (Tran et al. 2020). The types of interventions that aim to improve HRQL include physical fitness improvements, psychological interventions such as cognitive behaviour therapy (CBT) (Alsubaie et al. 2020), efforts to reduce psychological distress (Dalal et al. 2015); Tai Chi (Liu et al. 2022), and reducing social isolation (Love et al. 2021). Evidence suggests that interventions tailored to the individual may be more effective in improving HRQL than generic programmes (Kennedy et al. 2003; Koh et al. 2019). Furthermore, Linden et al. (1996) propose that psychosocial interventions show HRQL improvements as they provide nonspecific therapeutic benefits such as improving social support and fostering optimism.

CVD treatments can impact individuals' physical, mental, and social health and well‐being differently, and it is important to capture the effects of HRQL attributable to the condition (Seo et al. 2015; Tran et al. 2020). Some interventions have been implemented to enhance and maintain the HRQL of people living with CVD. However, a clear overview of these interventions for people with CVD and their effects on HRQL is missing. Likewise, no review to date has specifically examined the effects of interventions to improve HRQL among people living with CVD. Systematic reviews published elsewhere focus only on quality of life (Ni et al. 2022; Shepherd and While 2012; Su et al. 2020), but not HRQL. There are only two systematic reviews focused on HRQL: One examined the effects of nursing interventions on improving the HRQL among people with CVD (Yang et al. 2023), and the second review focused on the effects of cardiac rehabilitation (CR) on HRQL among people with coronary heart disease (Taylor et al. 2004).

## 2 The Review

### 2.1 Aim

To synthesise the evidence from randomised controlled trials on the effectiveness of interventions to improve HRQL in people living with cardiovascular disease.

### 2.2 Design

This systematic review followed the Joanna Briggs Institute (JBI) methodology to ensure a rigorous, accurate, and effective approach (Moher et al. 2020). The protocol was registered with Prospero CRD42022366687.

## 3 Method

### 3.1 Search Strategy

A preliminary search was conducted on CINAHL to identify keywords and other terms, using three broad categories (cardiovascular diseases AND health behaviour AND Health‐related quality of life). Keywords from identified records were then used to build a full search strategy including MeSH terms and adapted for each database (Appendix S1). The searches were conducted on eight databases (CINAHL, EMBASE, Medline, PsychINFO, Scopus, Web of Science, Cochrane library and JBI). Peer‐reviewed articles published in English were considered; there were no date limitations set, and the searches were completed by January 2024.

### 3.2 Selection and Inclusion Criteria

Articles identified in the database searches were uploaded into EndNote 20 (The EndNote Team 2013). Duplicate articles were identified and removed. Articles were transferred into to conduct a screening of the titles, abstracts, and full‐text articles Rayyan (Ouzzani et al. 2016). Two independent reviewers managed the screening.

The inclusion criteria were (1) population: adults aged over 18 diagnosed with CVD; (2) intervention: studies that included any type of intervention delivered in home, hospital or community that measured HRQL at baseline and follow‐up; (3) outcomes: HRQL; (4) study Design: Randomised controlled trial (RCT) with follow‐up at any time‐interval. This review also considered intervention studies that focused on only HRQL not quality of life (QOL) among people with CVD. ‘QOL is a broad concept covering all aspects of an individual life, whereas HRQL is a key patient‐reported outcome and an indicator of an individual's perception of their health including physical, social, and mental well‐being’ (Siqeca et al. 2022, 166).

This review excluded studies that focused on the following: children and adolescents, people with stroke, non‐ischaemic form of heart disease, at risk of developing CVD, and heart failure and those who used pharmacological or medical intervention. Two reviewers screened the title and abstract of each study independently, and differences of opinion were resolved through discussion. Articles deemed eligible for inclusion were independently blind‐screened at the full‐text level by two authors, with differences resolved through discussion.

### 3.3 Quality Appraisal and Data Extraction

Methodological quality and risk of bias of the included studies were apprised independently by two reviewers. Articles meeting the inclusion criteria were appraised using the JBI suite of quality appraisal checklists appropriate to each study (Moher et al. 2020) Data S1. Any disagreements between reviewers were discussed and resolved by the review team. Data were extracted using the JBI data extraction tools for RCTs (Piper 2019). Data extraction included context, country, study design, type of patient, sample size, type of intervention, sample size, demographic information, measurement, and HRQL. The primary reviewer extracted the data, then reviewed it by the other authors.

### 3.4 Data Synthesis

Due to the substantial heterogeneity among the selected studies in this systematic review, particularly in terms of the context of populations, measurement methods, comparators, and follow‐up periods, a meta‐analysis was not feasible. Instead, we applied the Synthesis Without Meta‐Analysis (SWiM) guidelines, as recommended for systematic reviews where meta‐analysis is not possible (Campbell et al. 2020). The key findings were synthesised narratively based on the type of intervention, length of follow‐up, and HRQL (Campbell et al. 2020).

## 4 Results

### 4.1 Study Inclusion

**FIGURE 1:**
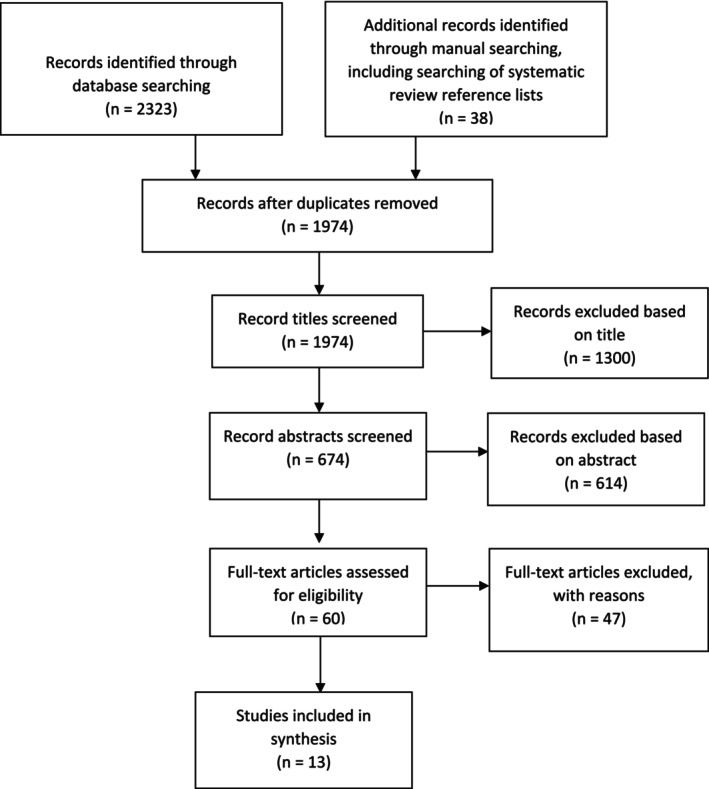
Study Selection and PRISMA flow diagram.

Figure 1 presents the selection of included studies. Initially, 2323 articles were identified from databases. Duplicates (*n* = 1974) were removed. Two reviewers conducted title and abstract screening independently. The remaining articles (*n* = 60) underwent full‐text screening. Forty‐seven studies were excluded for not meeting inclusion criteria. Thirteen studies (Arthur et al. 2002; Bailly et al. 2018; Batalik et al. 2020; Bravo‐Escobar et al. 2017; Hanssen et al. 2009; Hawkes et al. 2013; Hisam et al. 2022; Li et al. 2015; Lie et al. 2009; Pratesi et al. 2019; Seki et al. 2003; Smith et al. 2004; West et al. 2012) were retained for quality appraisal and included in the synthesis.

### 4.2 Methodological Quality of Included Studies

Two independent reviewers appraised the quality of each study (Appendix S2). Of the 14 included RCT studies (maximum quality score of 13), 12 studies scored 11 (Arthur et al. 2002; Bailly et al. 2018; Batalik et al. 2020; Bravo‐Escobar et al. 2017; Hanssen et al. 2009; Hawkes et al. 2013; Hisam et al. 2022; Li et al. 2015; Lie et al. 2009; Pratesi et al. 2019; Seki et al. 2003; Smith et al. 2004), and 1 study scored 9 (West et al. 2012).

### 4.3 Characteristics of Included Studies

The characteristics of the 13 studies are detailed in Appendix S3. Two studies were conducted in Norway (Hanssen et al. 2009; Lie et al. 2009), two studies in Canada (Arthur et al. 2002; Smith et al. 2004) and one study each in China (Li et al. 2015), France (Bailly et al. 2018), Czech Republic (Batalik et al. 2020), Pakistan (Hisam et al. 2022), the UK (West et al. 2012), Australia (Hawkes et al. 2013), Spain (Bravo‐Escobar et al. 2017), Italy (Pratesi et al. 2019), and Japan (Seki et al. 2003).

### 4.4 Population and Sample

The populations of the included studies reported comprised CVD (Bailly et al. 2018; Batalik et al. 2020; Li et al. 2015), ischemic heart diseases (Bravo‐Escobar et al. 2017), acute coronary syndrome (Hisam et al. 2022; Pratesi et al. 2019), myocardial infarction (Hanssen et al. 2009; Hawkes et al. 2013; Seki et al. 2003; West et al. 2012), and coronary artery bypass graft (CABG) (Arthur et al. 2002; Lie et al. 2009; Smith et al. 2004). Therefore, such heterogeneity within sampled populations and pooling participants with different types of heart conditions from the included studies prevented us from separating results for specific groups. Sample sizes of the included studies ranged from *n* = 28 (Bravo‐Escobar et al. 2017) up to *n* = 430 (Hawkes et al. 2013).

### 4.5 Summary Measure of HRQL

All of these HRQL measures were generic rather than being specific for CVD. The most commonly reported measure was a version of the Short‐Form QoL Questionnaire (SF36) (Reed 1998), which was used in the majority of studies (Arthur et al. 2002; Batalik et al. 2020; Bravo‐Escobar et al. 2017; Hanssen et al. 2009; Hawkes et al. 2013; Li et al. 2015; Lie et al. 2009; Pratesi et al. 2019; Seki et al. 2003; Smith et al. 2004; West et al. 2012), followed by the SF‐12 QoL Questionnaire (Hisam et al. 2022) and MacNew Heart Disease questionnaire (Hisam et al. 2022). The EuroQol EQ‐5D was used in one study (Bailly et al. 2018).

### 4.6 Intervention

The interventions used in all included studies were CR. They also used any core component of CR with different duration follow‐ups. There was variability in the comparator arms, and the most common comparators were considered for patients who received usual care, which limited external validity.

Two studies compared 6 months follow‐up hospital‐based CR with home‐based CR biweekly telephone monitoring, and both groups had access to nursing education, dietician, and psychologist counselling (Arthur et al. 2002; Smith et al. 2004). Two studies investigated hospital‐based CR with usual care without referral to rehabilitation and considered it as outpatients, either visiting a general practitioner or following up with a cardiologist at 6 and 12 months (Seki et al. 2003; West et al. 2012) education booklet for both groups.

Furthermore, one study looked at 12 weeks follow‐up home‐based telerehabilitation intervention and provided an instructive booklet, focusing on controlling diet, risk factors, and smoking compared with regular outpatient's hospital‐based CR (Batalik et al. 2020). Another study assessed monthly home‐based CR exercise program with hospital‐based for the initial six 6 months over receiving usual care outpatient CR in hospital for 12 months follow‐up (Pratesi et al. 2019). Home‐based CR telephone follow‐up providing information education and support based on patients' needs was evaluated with usual care physician outpatients (Hanssen et al. 2009). Additionally, home‐based CR combined with attending CR in hospital once a week compared with usual care undergoing CR in hospital. Both groups were educated on diet, medication, physical exercise, functioning heart, and returning to work (Bravo‐Escobar et al. 2017).

Three studies measured and monitored physical activity using a device, telerehabilitation device by the wrist heart rate monitor (Batalik et al. 2020), with a remote electrocardiographic monitoring device (Bravo‐Escobar et al. 2017) and pedometer‐self monitored and written in the exercise diary (Li et al. 2015). A few studies used other types of interventions to improve HRQL (refs). One study used a personalised telephone‐delivered health coaching intervention compared with usual care for 6 months (Hawkes et al. 2013). There was limited information reported on the content of the intervention and usual care. A further study examined the effectiveness of a progressively autonomous physical activity (PAPA) programme in comparison to standard supervised physical activity (SPA) (Bailly et al. 2018). This study did not provide further details on other elements of physical exercise. Most studies included in this review reported limited or no information on who delivered the intervention. However, in two studies, the intervention was delivered by experienced nurses (Li et al. 2015; Lie et al. 2009). Whilst three studies used a multidisciplinary team to deliver the intervention (Batalik et al. 2020; Seki et al. 2003; West et al. 2012). Less attention was given to psychological and social components in the included studies. However, one study (Lie et al. 2009) investigated home‐based psychological support with no exercise component. This consisted of structured information at 2‐ and 4‐weeks post‐surgery in comparison with receiving standard discharge care that did not have a brief conversation with the nurse/doctor. Also, three studies provided a psychological support for intervention and control groups (Arthur et al. 2002; Bravo‐Escobar et al. 2017; Hisam et al. 2022; Smith et al. 2004).

### 4.7 Review Findings

#### 4.7.1 Physical Well‐Being

Most studies reported improvements in the physical component of HRQL. The score of physical components improved in both groups (*p* < 0.0001) (Arthur et al. 2002); home CR had a better score compared to the hospital group (51.2 6.4 vs. 48.6 7.1, *p* < 0.05). Interestingly, in one study (Lie et al. 2009) where the tested intervention had no exercise component, the significant difference appeared in the physical domain (74 vs. 70 *p* < 0.01). The significant improvement was observed in the control and intervention groups at 6 weeks and 6 months, but no significant difference among the groups (Bailly et al. 2018). However, the health utility component improved merely in the intervention group at 12 months (0.828 vs. 0.882, *p* = 0.4) (Bailly et al. 2018). Three trials (Batalik et al. 2020; Hawkes et al. 2013; Hisam et al. 2022) used telephone to deliver CR. The physical composite scores were compared at 12 weeks and indicated an improvement in the physical composite score (23.4 vs. 25.9 *p* = 0.02) (23.7 vs. 26.5 *p* = 0.04) in the control group and intervention home‐telerehabilitation group, respectively (Batalik et al. 2020). The mobile group showed greater physical activity compared to the control group at 12 weeks (48.93 vs. control 43.87, *p* < 0.001) and 6 months (53.52 vs. 46.82 *p* < 0.001) (Hisam et al. 2022). The physical domain changed significantly over the 6 months only in the intervention group (Hawkes et al. 2013). Also, home CR intervention improved the physical component significantly in the intervention group from baseline to 3 months (Li et al. 2015). Likewise, the physical domain in both groups at 6 months improved significantly (7.31 vs. 4.98 *p* = 0.039) but the advantage did not maintain at 12 or 18 months (Hanssen et al. 2009). This study also indicated that people aged 70 years old and over had a better improvement in physical function at 18 months (*p* = 0.025) (Hanssen et al. 2009). In a smith et al. study, habitual physical activity was examined; the intervention group had a higher score compared to the control group at 12 months (232.6 vs. 170 *p* = 0.005) (Smith et al. 2004). One study reported that by 6 months, physical composite scores had significantly increased from baseline (*p* < 0.0.5) in the intervention group only, with no differences among the groups (Seki et al. 2003). The significant differences in the physical domain were not reported in the remaining trials (Pratesi et al. 2019; West et al. 2012), among the intervention and control groups, and no differences by treatment arms over the whole follow‐up (Pratesi et al. 2019). One study did not report specific data on physical domains (Bravo‐Escobar et al. 2017).

#### 4.7.2 Psychological Well‐Being

Mental health scores in the intervention and control groups improved significantly in four studies (Arthur et al. 2002; Batalik et al. 2020; Hawkes et al. 2013; West et al. 2012). However, these changes did not achieve statistical significance at three or 6 months (Arthur et al. 2002) or 12 weeks (Batalik et al. 2020). Another study reported the lowest score in the intervention group of patients under 50 years old, suggesting an adverse effect for this specific age group (Hanssen et al. 2009). While a significant improvement was indicated at 6 weeks for the intervention and control groups, by 6 months, the significant improvement was observed only in the intervention group, with no differences between groups at each time point (Lie et al. 2009). Mental component scores improved significantly in the intervention group at each time (*p* < 0.001) (Hisam et al. 2022). No significant changes were observed over the 6 months in the control groups, while significant improvements were observed for the intervention group (Seki et al. 2003). The results for psychological well‐being were not reported in two studies (Bailly et al. 2018; Bravo‐Escobar et al. 2017), and the effects of the intervention on mental health scores were not reported for either group in three studies (Li et al. 2015; Pratesi et al. 2019).

#### 4.7.3 Social Well‐Being

The social domain of well‐being of the intervention home group at both time points achieved a higher score for each sub‐scale compared to the hospital group; therefore, this group had a better sense of belonging support (Arthur et al. 2002). Likewise, one specific study indicated a positive score in the CR home group compared to the hospital group; however, there was a decline in available support in the home group at 12 months after discharging from CR (Smith et al. 2004). One study (Hisam et al. 2022) found improvement in social function scores over time but no significant difference between groups. The social domain was not reported in four studies (Bailly et al. 2018; Bravo‐Escobar et al. 2017; Hawkes et al. 2013; Pratesi et al. 2019). Social function was reported in other studies over time, but there were no significant differences between the two groups (Batalik et al. 2020; Hanssen et al. 2009; Li et al. 2015; Lie et al. 2009; Seki et al. 2003; West et al. 2012).

#### 4.7.4 Overall HRQL

In general, all included studies regardless of the type of CR and instrument, individuals who received CR improved overall HRQL compared to those who received standard care. One study (Arthur et al. 2002) presented that both home and hospital CR improved HRQL outcomes. Likewise, the improvement in HRQL was observed in both groups, but in the supervised group, the score was reduced by 1 year (Bailly et al. 2018). In another two studies (Batalik et al. 2020; Bravo‐Escobar et al. 2017), HRQL improved substantially, but there were no significant differences between groups. Five studies did not report the overall score of HRQL (Hawkes et al. 2013; Lie et al. 2009; Pratesi et al. 2019; Seki et al. 2003; West et al. 2012).

## 5 Discussion

Cardiovascular disease (CVD) is associated with both physical and emotional challenges that significantly impact health‐related quality of life (HRQL). HRQL measures offer a structured way to evaluate the effectiveness of different interventions using valid and reliable scales. However, the relationship between specific interventions and improvements in HRQL among people living with CVD remains insufficiently explored in the literature. Understanding how various interventions influence HRQL on a comparable scale would provide critical insights for policymakers in prioritising health interventions and optimising patient outcomes. This review identified 2323 articles, of which 14 met the inclusion criteria. The findings suggest that, overall, interventions contributed to improvements in HRQL, though the extent of benefits varied across domains.

CR emerged as an effective, convenient, and adaptable intervention, delivered through in‐person sessions, smartphone applications, or web‐based platforms. This flexibility likely contributes to its high acceptability, as it enables participants to maintain communication with healthcare professionals and receive tailored, individualised support. CR had the most pronounced impact on the physical domain of HRQL, with improvements in physical activity, fitness levels, and exercise capacity leading to important physiological adaptations that benefit cardiovascular health. Notably, even in a study that lacked a structured exercise component (Lie et al. 2009), CR still produced positive effects in physical outcomes, underscoring its broad benefits. Additionally, CR played a critical role in post‐cardiac event recovery, supporting sustained engagement in physical activity, which in turn enhanced HRQL and overall health (Hurdus et al. 2020). These findings align with prior research (Bakker et al. 2020), highlighting CR's role in reinforcing positive physical activity habits, ultimately leading to better physical conditioning and improved HRQL.

CR also demonstrated positive effects on the psychological domain, though improvements in mental health outcomes were generally less pronounced than those observed in physical health. However, the strong link between physical and psychological well‐being suggests that the physiological benefits of CR may contribute indirectly to better mental health. This aligns with previous findings (Choo et al. 2018), which reported reductions in anxiety and depression scores, confirming CR's effectiveness in enhancing psychological well‐being among CVD patients.

## 6 Limited Evidence on the Social Domain

Despite the recognised importance of social well‐being in HRQL, few studies in this review examined HRQL within the social domain. Home‐based CR demonstrated some benefits in this area, possibly due to the individualised nature of home‐based programs, which may foster stronger patient engagement and autonomy. However, only one study (Arthur et al. 2002) directly compared social HRQL outcomes between intervention and control groups, making broader comparisons difficult. Nevertheless, existing evidence (Staniute et al. 2013) suggests that CR inherently incorporates a social support component, which may mitigate the negative effects of CVD‐related functional limitations and enhance social well‐being.

## 7 Future Directions

While CR interventions show promising effects on HRQL, future research should adopt a broader and more integrated approach to address the physical, psychological, and social needs of individuals living with CVD in a broader context. Further studies should explore the underlying mechanisms driving the effectiveness of CR, as well as its potential for optimisation through nursing interventions and rehabilitation plans. Standardising HRQL assessment tools across studies would enable better comparisons and data synthesis, strengthening the evidence base for clinical and policy decision‐making. More studies should explore the social dimensions of HRQL, as social support plays a crucial role in the long‐term well‐being of people living with CVD. By refining intervention strategies and expanding research into the multidimensional aspects of HRQL, healthcare providers can develop more targeted and holistic approaches to improve the well‐being of individuals living with CVD, ultimately enhancing their long‐term health outcomes and quality of life.

## 8 Strengths and Limitations

This systematic review employed a robust methodology and a comprehensive search strategy. The JBI framework (Moher et al. 2020) was chosen for its rigorous approach to establishing inclusion and exclusion criteria, ensuring the selection of relevant studies. Additionally, this framework provides guidance on selecting the most appropriate critical appraisal tool to assess study quality, enhancing the clarity, trustworthiness, and consistency of the results. Despite heterogeneity in study outcomes, comparative measures, and follow‐up periods, a narrative synthesis was conducted. This approach, as recommended by Aromataris et al. (2017), is suitable when meta‐analysis is not feasible.

The studies included in this review used validated instruments to measure outcomes, and eight databases were searched to ensure comprehensive coverage and reduce the risk of missing eligible studies. However, methodological limitations in the included studies may introduce biases, affecting the reliability of evidence on the efficacy of the intervention for improving HRQL. Statistically significant improvements in HRQL were not observed across all studies. While all included studies used validated instruments to assess HRQL, the choice of instruments varied, making direct comparisons challenging and limiting the ability to translate findings into clear recommendations. Additionally, the variability in the rate of recurrence of the interventions has contributed to inconsistent HRQL outcomes.

The heterogeneity in measurement tools further prevents the feasibility of conducting a meta‐analysis. Moreover, control groups were not included in 27% of the studies, restricting the ability to perform robust pre‐and post‐intervention comparisons. The lack of data on social, mental health outcomes, as well as overall HRQL, further complicates drawing meaningful conclusions about effective interventions for people living with CVD. To address these limitations, future research should define a clear intervention protocols and adopt standardised outcome measures. This would enable subgroup analyses and data pooling, allowing for a deeper understanding of the evidence. A more standardised approach would strengthen the reliability of conclusions and improve the overall quality of evidence in this field.

## 9 Conclusion and Implication

CVD significantly impacts physical and emotional well‐being, leading to a reduced HRQL. This highlights the need for effective interventions that can mitigate these negative effects and enhance overall quality of life. By systematically evaluating the impact of interventions on HRQL, policymakers and healthcare providers can make informed decisions about prioritising and optimising health interventions for individuals living with CVD.

Despite the recognised burden of CVD on HRQL, the relationship between specific interventions and HRQL improvements remains underexplored in the literature. This review identified CR as a commonly used and highly effective intervention for improving HRQL, particularly in the physical and psychological domains. The physical benefits of CR were the most pronounced, with improvements in physical activity, fitness, and exercise capacity observed even in studies that did not include structured exercise components. Although the psychological benefits were generally less substantial than the physical improvements, CR contributed to reductions in anxiety and depression, demonstrating its potential to enhance mental HRQL post‐intervention. However, the social domain of HRQL remains underexamined. The limited evidence available suggests that home‐based CR interventions may offer social benefits, likely due to their individualised approach and embedded social support mechanisms. Given the role of social interactions in long‐term health outcomes, nurses and healthcare professionals delivering CR programmes could consider enhancing the social support component of these interventions. Additionally, more research is needed to develop and apply standardised social domain measures, ensuring a comprehensive assessment of HRQL outcomes.

## Conflicts of Interest

The authors declare no conflicts of interest.

## Supporting information

Appendices S1–S3.

Data S1.

## Data Availability

Data sharing not applicable to this article as no datasets were generated or analysed during the current study.
