# Supplementary material for: The Effects of Interventions on Health‐Related Quality of Life of People Living With Cardiovascular Disease: A Systematic Review
Source: J Clin Nurs. 2025 Apr 28;34(6):2067–74. doi: 10.1111/jocn.17770 (PMC12125534; doi:10.1111/jocn.17770)
Supplement: Supplementary file 1 — Appendices S1–S3. [file JOCN-34-2067-s001.docx]

**Appendix S1: Search Strategy**

**CINAHL:** cardiovascular health problems OR ( heart or cardiac or cardiovascular or coronary ) OR heart disease OR CVD OR (MM "Cardiovascular Diseases") AND ( (MM health behavior) or (MM life style) ) OR best practice* OR Strategies OR evaluation OR lifestyle OR Therap* OR therapy OR intervention OR lifestyle intervention* AND ( (MM "Quality of life") OR (MM'"Health status") OR (MM "Self Care") ) OR ( well-being or wellbeing or well being ) OR ( "health related quality of life" or hrqol or qol ) OR psychosocial factors OR life quality

**Medline**: cardiovascular health problems OR ( heart or cardiac or cardiovascular or coronary ) OR heart disease OR CVD OR (MM "Cardiovascular Diseases") AND ( (MM health behavior) or (MM life style) ) OR best practice* OR Strategies OR evaluation OR lifestyle OR Therap* OR therapy OR intervention OR lifestyle intervention* AND ( (MM "Quality of life") OR (MM'"Health status") OR (MM "Self Care") ) OR ( well-being or wellbeing or well being ) OR ( "health related quality of life" or hrqol or qol ) OR psychosocial factors OR life quality

**PsychInfo :** cardiovascular health problems OR ( heart or cardiac or cardiovascular or coronary ) OR heart disease OR CVD OR (MM "Cardiovascular Diseases") AND ( (MM health behavior) or (MM life style) ) OR best practice* OR Strategies OR evaluation OR lifestyle OR Therap* OR therapy OR intervention OR lifestyle intervention* AND ( (MM "Quality of life") OR (MM'"Health status") OR (MM "Self Care") ) OR ( well-being or wellbeing or well being ) OR ( "health related quality of life" or hrqol or qol ) OR psychosocial factors OR life quality

**Embase**: (('cardiovascular disease'/exp OR 'angiocardiopathy' OR 'angiocardiovascular disease' OR 'cardiovascular complication' OR 'cardiovascular disease' OR 'cardiovascular diseases' OR 'cardiovascular disorder' OR 'cardiovascular disturbance' OR 'cardiovascular lesion' OR 'cardiovascular syndrome' OR 'cardiovascular vegetative disorder' OR 'complication, cardiovascular' OR 'disease, cardiovascular' OR 'major adverse cardiovascular event' OR cardiovascular) AND health AND problems OR cardiac OR cardiovascular OR coronary OR heart) AND disease OR cvd AND health behaviors'/exp OR 'lifestyle'/exp OR 'best practice*' OR strategies OR evaluation OR lifestyle OR therap* OR therapy OR intervention OR lifestyle AND quality of life'/exp OR 'health status'/exp OR 'self care'/exp OR 'wellbeing'/exp OR 'well-being' OR 'well being' OR 'health related quality of life' OR hrqol OR qol OR 'psychosocial factors' OR 'life quality' AND #4 AND [embase]/lim NOT ([embase]/lim AND [medline]/lim) AND 'cardiovascular disease'/dm AND 'human'/de AND ([adult]/lim OR [aged]/lim OR [middle aged]/lim OR [very elderly]/lim OR [young adult]/lim) AND 'article'/it AND [english]/lim

**Scopus** ( ( ( TITLE-ABS-KEY ( *{cardiovascular health problems}*  OR  *heart*  OR  *cardiac*  OR  *cardiovascular*  OR  *coronary*  OR  *{heart disease}*  OR  *cvd*  OR  *{Cardiovascular Diseases}* ) )  AND  ( TITLE-ABS-KEY ( *{health behaviors}*  OR  *lifestyle*  OR  *{best practice*}*  OR  *strategies*  OR  *evaluation*  OR  *{life style}*  OR  *therap**  OR  *therapy*  OR  *intervention*  OR  *lifestyle* ) )  AND  ( TITLE-ABS-KEY ( *{quality of life}*  OR  *{health status}*  OR  *{self care}*  OR  *wellbeing*  OR  *{well-being}*  OR  *{well being}*  OR  *{health related quality of life}*  OR  *hrqol*  OR  *qol*  OR  *{psychosocial factor}*  OR  *{life quality}* ) ) )  AND NOT  ( TITLE-ABS-KEY ( *{pharmocological intervention}*  OR  *{medical procedure}*  OR  *{risk factors}*  OR  *{contributing factors}*  OR  *{predisposing factors}* ) ) )  AND  ( KEY ( *{cardiovascular diseases}*  AND  *adults* ) )  AND  ( LIMIT-TO ( DOCTYPE ,  *"ar"* ) )  AND  ( LIMIT-TO ( EXACTKEYWORD ,  *"Human"* ) )  AND  ( LIMIT-TO ( LANGUAGE ,  *"English"* ) )  AND  ( LIMIT-TO ( SRCTYPE ,  *"j"* ) )

**Web 0f Science** : cardiovascular health problems OR ( heart or cardiac or cardiovascular or coronary ) OR heart disease OR CVD OR (MM "Cardiovascular Diseases") (Topic) AND ( (MM health behavior) or (MM life style) ) OR best practice* OR Strategies OR evaluation OR lifestyle OR Therap* OR therapy OR intervention OR lifestyle intervention* (Topic) AND ( (MM "Quality of life") OR (MM'"Health status") OR (MM "Self Care") ) OR ( well-being or wellbeing or well being ) OR ( "health related quality of life" or hrqol or qol ) OR psychosocial factors OR life quality  AND ALL=(Adult AND Cardiovascular Disease AND English NOT ( pharmacological intervention OR medical procedure ) NOT ( risk factors or contributing factors or predisposing factors ) ) and 1.37 Cardiology - General (Citation Topics Meso) and Article (Document Types) and Cardiac Cardiovascular Systems (Web of Science Categories)

**Appendix S2: Methodological quality of studies**

| Citation | Q1 | Q2 | Q3 | Q4 | Q5 | Q6 | Q7 | Q8 | Q9 | Q10 | Q11 | Q12 | Q13 | Score |
| --- | --- | --- | --- | --- | --- | --- | --- | --- | --- | --- | --- | --- | --- | --- |
| Arthur et al. (2022) | Y | Y | Y | Y | U | U | Y | Y | Y | Y | Y | Y | Y | 11/13 |
| Bailly, L., (2018). | Y | Y | Y | Y | U | U | Y | Y | Y | Y | Y | Y | Y | 11/13 |
| Batalik, L. (2020). | Y | Y | Y | Y | U | U | Y | Y | Y | Y | Y | Y | Y | 11/13 |
| Bravo-Escobar, R., (2017). | Y | Y | N | Y | N | Y | Y | Y | Y | Y | Y | Y | Y | 11/13 |
| Hanssen et al. (2009) | Y | Y | Y | Y | U | U | Y | Y | Y | Y | Y | Y | Y | 11/13 |
| Hawkes Anna, L. (2013). | Y | Y | Y | Y | U | U | Y | Y | Y | Y | Y | Y | Y | 11/13 |
| Hisam, A., Ul . (2022). | Y | Y | Y | Y | U | U | Y | Y | Y | Y | Y | Y | Y | 11/13 |
| Lie et al., 2009 | Y | Y | Y | Y | U | U | Y | Y | Y | Y | Y | Y | Y | 11/13 |
| Li et al., 2015 | Y | Y | Y | Y | U | U | Y | Y | Y | Y | Y | Y | Y | 11/13 |
| Pratesi, A.(2019). | Y | Y | Y | Y | U | U | Y | Y | Y | Y | Y | Y | Y | 11/13 |
| Seki et al. (2003) | Y | Y | Y | Y | U | U | Y | Y | Y | Y | Y | Y | Y | 11/13 |
| Smith et al (2004) | Y | Y | Y | Y | U | U | Y | Y | Y | Y | Y | Y | Y | 11/13 |
| West Robert(2012). | Y | Y | Y | Y | N | Y | Y | Y | U | U | U | Y | Y | 9/13 |

Y, yes; N, no; U, unclear; NA; not applicable

1.Was true randomization used for assignment of participants to treatment groups?

2. Was allocation to treatment groups concealed?

3. Were treatment groups similar at the baseline?

4. Were participants blind to treatment assignment?

5. Were outcomes assessors blind to treatment assignment?

6. Were outcomes assessors blind to treatment assignment?

7. Were treatment groups treated identically other than the intervention of interest?

8. Was follow up complete and if not, were differences between groups in terms of their follow up adequately described and analyzed?

9. Were participants analysed in the groups to which they were randomized?

10. Were outcomes measured in the same way for treatment groups?

11. Were outcomes measured in a reliable way?

12.Was appropriate statistical analysis used?

13. Was the trial design appropriate, and any deviations from the standard RCT design (individual randomisation, parallel groups) accounted for in the conduct and analysis of the trial?

**Appendix S3: Characteristics of included studies for methodological review**

| Study | Country | Participants characteristics sample size | Intervention description | Outcome measured | Description main results |
| --- | --- | --- | --- | --- | --- |
| Arthur et al. (2022) | Canada | Post CABG patients  N= 242  Age mean was  63.35 years and 81% were male | Hospital-based group  Exercise classes 3x per  week for 6 months 40min  aerobic exercise. Access to other elements of cardiac  rehabe e.g. dietician  psychologist | The primary outcome was peak exercise capacity and secondary outcomes was HRQL. | The study groups had similar demographic and health profiles at baseline. Peak V˙ O2 improved significantly in both groups after 6 months of exercise training; 36% in the Hosp group (1222.1 269.0 mL·min1 to 1497.2 594.3 mL·min1 ; P 0.0001) and 31% in the Home group (1260.3 306.5 mL·min1 to 1433.4 589.7 mL·min1 ; P 0.05). The Home group reported greater total social support than the Hosp group at 3 (36.2 4.5 vs 34.0 6.7; P 0.0001) and 6 months (36.0 4.9 vs 34.6 6.4; P 0.05). The Home group demonstrated a greater improvement in health-related quality of life (physical) by 6 months in comparison to the Hosp patients (51.2 6.4 vs 48.6 7.1; P 0.004). |
| Bailly, L., (2018). | France | CVD patients  age mean was 62.5 and 95.5% were male.  N= 50 | Progressively autonomous physical activity (PAPA) program or to a standard supervised physical activity (SPA)  program. The SPA group had two supervised sessions per week over 5 months. PAPA group had one session per  week and support to aid habit formation (written tips, exercise program, phone call). | The outcomes were HRQL, Physical functioning ,and healthcare costs. | Mobility, usual activities and discomfort improved significantly in both group (T6). One year later, EQ-5D  utility score was improved in the PAPA group only. Total health care consumption in the intervention group  decreased, from a mean of 4097 euros per year before intervention to 2877 euros per year after (p = 0.05),  compared to a health care consumption of 4087 euros and 4180 euros per year, in the total population of patients  (N = 1891) from the health insurance company. The incremental cost effectiveness ratio was 10,928 euros per QALYs. |
| Batalik, L. (2020). | Czech Republic | cardiac rehabilitation patients, age mean was 57 and 85% were male.  N=56 | cardiac rehabilitation patients were randomized into a 12-week regular outpatient training group (ROT) and  interventional home-based telerehabilitation group (ITG). For both groups, the intensity of the training was prescribed to be performed  at 70% to 80% of heart rate reserve for 60 minutes, 3 times a week. The ITG patients started their training with a wrist heart rate  monitor in their home environment. These patients received feedback once a week, reﬂecting data uploaded on the internet  application. The ROT patients performed their exercise under the direct supervision of a physical specialist in a regular outpatient  clinic. | Primary outcomes was physical ﬁtness.  Secondary outcome were HRQL and training adherence. | Fifty-one patients comleted the intervention (91%); no serious adverse events were recorded. Physical ﬁtness expressed  as peak oxygen uptake showed signiﬁcant improvement (P< .001) in ROT group from 23.4± 3.3 to 25.9± 4.1 mL/kg/min and  (P< .01) in ITG group from 23.7± 4.1 to 26.5± 5.7 mL/kg/min without signiﬁcant between-group differences after 12 weeks of  intervention. The training adherence between groups was similar. |
| Bravo-Escobar, R., (2017). | Spain | Patients with coronary artery disease.  N=28 | The group undergoing traditional cardiac rehabilitation in hospital (control group) and 14 were assigned to the homebased  mixed surveillance programme (experimental group). The patients in the experimental group went to the cardiac  rehabilitation unit once a week and exercised at home, which was monitored with a remote electrocardiographic  monitoring device (NUUBO®). The in-home exercises comprised of walking at 70% of heart rate reserve during the first  month, and 80% during the second month, for 1 h per day at a frequency of 5 to 7 days per wee | The HRQL and cardiovascular complications. Measurement were taken at baseline and 4-6 weeks months. | No significant differences were observed between the traditional cardiac rehabilitation group and the homebased  with mixed surveillance group for exercise time and METS achieved during the exertion test, and the recovery  rate in the first minute (which increased in both groups after the intervention). The only difference between the two  groups was for quality of life scores (10.93 [IC95%: 17.251, 3.334, p = 0.007] vs −4.314 [IC95%: −11.414, 2.787; p = 0.206]).  No serious heart-related complications were recorded during the cardiac rehabilitation programme. |
| Grady, K. L., (2014). | USA | HF patients  Age mean was  63.6 years and 52% were male  N=902 | Patients who were randomized to self-management participated in 18 two hour group counseling sessions spread over a 1-year time period that coached them in the development of self-management skills and problem solving and were provided with HF educational information intended to improve adherence with their medical regimen and ultimately improve HRQOL.  During group meetings, participants received one of the 18 HF educational materials and were taught self-management skills such as self-monitoring (e.g., regarding sodium consumption), environmental restructuring, elicitation of support from family, and cognitive restructuring.  The enhanced education control group received the same 18 HF educational materials, per mail, as the self-management intervention group at the same pre- specified time intervals during a 1-year time period, followed by a telephone call to ensure receipt, check comprehension, and answer questions about the information. | HRQL | Overall, study participants’ HRQOL  improved over time. However, no signiﬁcant differences in  HRQOL domain were detected between treatment groups  at baseline or across time (p [ 0.05). Subgroup analyses  demonstrated no differences by treatment arm for change  in HRQOL from baseline to 3 years later. |
| Hanssen et al. (2009) | Norway | Post MI Age mean 60.2 years M 80.9%, F 19.1%  N=288 | elephone follow-up and open telephone line. Providing information education and support in accordance with patients’ needs. Calls weekly for first 4 weeks then on weeks 6, 8, 12 and 24 | HRQL | There were significant improvements over time on most dimensions of health-related quality of life in both the  intervention and control group to US norm population levels on most SF-36 dimensions and summary scores. The intervention group showed no overall significant improvement beyond six months in the physical or mental summary scores, but there was a significant effect for those aged 70 or above. Although there was a promising effect for rehospitalisation due to chest pain, no  significant differences were found between the groups on the secondary endpoints after six months. |
| Hawkes Anna, L. (2013). | Australia | Post MI patients, mean age was 60.5 and 79% male. N=430 | participants received up to 10 × 30 min scripted telephone health coaching sessions. This included an introductory session to explain the program and what was expected of the participant, followed by three weekly sessions, three fortnightly sessions and four monthly sessions over 6 months to assist with CHD risk factor management. During the intervention period, participants were guided through a series of steps beginning with an assessment of their CHD risk factor profile at the commencement of the program followed by feedback on their profile. | HRQL | Physical HRQoL was lower in participants who: were older (p<0.001); were unemployed (p=0.03); had lower  baseline physical and mental HRQoL scores (p<0.001); had lower confidence levels in meeting sufficient physical activity recommendations (p<0.001); had no intention to be physically active in the next six months (p<0.001); and were more sedentary (p=0.001). Mental HRQoL was lower in participants who: were younger (p=0.01); had lower baseline mental HRQoL (p<0.001); were more sedentary (p=0.01) were depressed (p<0.001); and had lower social support (p=0.001). |
| Hisam, A., Ul . (2022). | Pakistan | Patients with acute coronary syndrome Mean age was 52 and 785 of male  N=160 | The intervention group received the Mobile Cardiac rehabilitation, a medically supervised cardiac rehabilitation program in addition to standard care. The first phase included individualised psychotherapy during the hospital stay. The second phase included diurnal mobile texting of standardised messages about healthy lifestyle changes through a specially developed app. The control group received standard post-ACS care. | HRQL | At baseline, 160 patients (80 in each group; mean age 52.66±8.46 years; 126 male, 78.75%) were  recruited, of which 121(75.62%) continued and were analysed at 12-weeks and 119(74.37%) at 24-weeks.  The mean SF-12 physical component score significantly improved in the MCard group at 12 weeks follow-up  (48.93 vs control 43.87, p<.001) and 24 weeks (53.52 vs 46.82 p<.001). The mean SF-12 mental component  scores also improved significantly in the MCard group at 12 weeks follow-up (44.84 vs control 41.40,  p<.001) and 24 weeks follow-up (48.95 vs 40.12, p<.001). At 12-and 24-week follow-up, all domains of  MacNew QLMI (social, emotional, physical and global) were also statistically significant (p<.001) improved  in the MCard group, unlike the control group |
| Lie et al., 2009 | Norway | Patients with post CABG  mean age was 62 years and male 89%  N=185 | Home-based psycho-educative intervention, consisting of structured information and psychological support for the topics angina symptoms, medications, sexuality, anxiety and depression. 1 h home visits at 2 and 4 weeks post-surgery *n* = 92 | HRQL | Significant improvements were found in both groups for the majority of subscales of HRQoL at 6-week and 6-month follow-up. However, these improvements did not differ significantly between the groups. Compared to the general population, significant differences (*P* < 0.05) were found for the SF-36 subscales: role physical, role emotional and bodily pain. |
| Lie et al., 2015 | China | Patients with CVD, mean age was 80.6 and male was 84%  N=61 | The intervention group received  a 12-week, home-based, exercise rehabilitation program  as well as regular follow-up led by the advanced practice nurse who had received  specific rehabilitation nursing training. | HRQL and LVEF | After 12 weeks, the intervention group showed significant improvements in HRQOL (physical functioning, role-physical, bodily pain, and vitality; p < .05) as well as on the Senior Fitness Test (chair stands, arm curls, Timed Up and Go, and 6-minute walk distance; p < .05); there was no significant improvement in LVEF (p = .56). |
| Pratesi, A.(2019). | Italy | Patients with acute coronary syndrome Mean age was 81 and 66.5% male  N=197 | Patients in the intervention were prescribed a standardised set of homebased  exercises with centre-based monthly reinforcements for the first 6 months. The main (peak oxygen consumption) and  three secondary outcome measures (distance walked in 6 minutes, inferior limbs peak 90  Torque strength, health-related  quality of life) were assessed at baseline, at random assignment and at 6 and 12-month follow-ups with the cardiopulmonary  exercise test, 6-minute walking test, isokinetic dynamometer and the Short Form-36 questionnaire, respectively. | HRQL, adherence and hospitalisation rate | Both groups obtained a significant and similar improvement from baseline to the end of the 4-week  cardiac rehabilitation programme in the three functional outcome measures. However, at univariable and age and gender- adjusted analysis of variance for repeated measures, changes from random assignment to 6 or 12-month follow-up in any outcome measure were similar in the C and T groups. |
| Seki et al. (2003) | Japan | RCT two group post MI,CABG Age> 65 years ;mean, 70years M100  N=38 | In the intervention group, patients participated in an outpatient phase III CR program for 6 months. The program included an exercise session and exercise prescription, and a dietary and educational program. The weekly supervised exercise session at the clinic consisted of approximately  20 min of warm-up exercises including stretching and calis- thenics, followed by 20–30 min of continuous upright aerobic and dynamic exercise (various combinations of walking, bicycling, jogging, and other activities) quantita- tively and light isometric exercise, such as hand weights, and approximately 20 min of cool-down stretching and calisthenics. The intensity of exercise was prescribed. | The outcome was HRQL. | After 6 months,  in the intervention group, scores of bodily pain, general health, vitality and mental health of SF-36 improved  signiﬁcantly compared with baseline. State anxiety scores also improved signiﬁcantly (p<0.01), but depression scores were not improved. In the control group, none of the parameters  signiﬁcantly  changed. |
| Smith et al (2004) | Canada | Patients with CABG with mean age was 63 and 79% male.  N=222 | Patients were followed-up 12 months after discharge from a RCT of 6  months of monitored ‘Home’ versus supervised ‘Hospital’ CR after CABG. | The primary outcome was exercise capacity and secondary outcomes were HRQL and SS. | One hundred and ninety-eight patients (89.2%), 102 ‘Hospital’ and 96 ‘Home’, returned for follow-up 12-months  after discharge from CR. Both groups had similar medical and socio-demographic characteristics. Peak VO2 declined in  ‘Hospital’ but was sustained in ‘Home’ patients 12 months after discharge from CR (P = 0.002). Physical HRQL was higher  in the ‘Home’ group at the 12-month follow-up (P < 0.01). Mental HRQL showed general, minor deterioration over time in  both groups (P = 0.019). Twelve months after discharge from CR, physical and mental HRQL remained higher than at entry  to CR in both groups. ‘Home’ patients had higher habitual physical activity scores compared to ‘Hospital’ patients. |
| West Robert(2012). | UK | N=331 |  | The primary outcome measure was all-cause mortality at 2 years. The secondary measures were morbidity, health service use, health-related quality of life, psychological general well-being and lifestyle cardiovascular risk factors at 1 year. Measurement were taken at baseline, 12 months | There were no significant differences between patients referred to rehabilitation and controls in mortality at 2 years (RR 0.98, 95% CI 0.74 to 1.30) or after 7–9 years (0.99, 95% CI 0.85 to 1.15), cardiac events, seven of eight domains of the health-related quality of life scale (‘Short Form 36’, SF36) or the psychological general well-being scale. Rehabilitation patients reported slightly less physical activity. No differences between groups were reported in perceived overall quality of cardiac aftercare. Data from the ‘elective’ hospitals comparison concurred with these findings |
